# Corticosteroid treatment for acute hydrocephalus in neurosarcoidosis: a case report

**DOI:** 10.1186/s13256-024-04359-9

**Published:** 2024-02-13

**Authors:** Edoardo Dalmato Schilke, Giulia Remoli, Claudia Cutellé, Claudia Balducci, Diletta Cereda, Maria Letizia Fusco, Lucio Tremolizzo, Carlo Ferrarese, Ildebrando Appollonio, Maura Frigo

**Affiliations:** 1 Neurology Department, Fondazione IRCCS San Gerardi Dei Tintori, San Gerardo Hospital, Monza. Via G.Pergolesi, 33, 20900 Monza, Italy; 2 School of Medicine and Surgery and Milan Centre for Neuroscience (NeuroMI), University of Milano-Bicocca, Milan, Italy

**Keywords:** Neuroimmunology, Neurosarcoidosis, Obstructive hydrocephalus, Neurosurgery in sarcoidosis

## Abstract

**Background:**

Neurosarcoidosis occurs symptomatically in 5–10% of patients with sarcoidosis, and hydrocephalus is a rare complication of neurosarcoidosis, with either acute or subacute onset and presenting symptoms related to increased intracranial pressure. It represents a potentially fatal manifestation with a mortality rate of 22% (increased to 75% in case of coexistence of seizures) that requires a prompt initiation of treatment. High-dose intravenous corticosteroid treatment and neurosurgical treatment must be considered in all cases of neurosarcoidosis hydrocephalus.

**Case presentation:**

Here we present a case of hydrocephalus in neurosarcoidosis, complicated by generalized seizures, in a 29-year-old Caucasian male patient treated with medical treatment only, with optimal response.

**Conclusion:**

Since neurosurgery treatment can lead to severe complications, this case report underlines the possibility to undergo only medical treatment in selected cases. Further studies are needed to stratify patients and better identify those eligible for only medical approach.

## Background

Sarcoidosis is a multisystemic inflammatory disorder characterized by a cell-mediated granulomatous response to as-yet unidentified antigens [1]. Clinically recognizable nervous system involvement was reported in 5–10% of all patients, although clinically occult neurosarcoidosis (NS) was identified during autopsy in up to 15–25% of patients. Conversely, 10–20% of patients with NS do not present systemic sarcoidosis (isolated NS) [2, 3].

Noncaseating granulomas are made of epithelioid macrophages and CD4^+^ T helper cells, surrounded by a ring of fibroblasts and B cells, and CD8^+^ cytotoxic T cells at the periphery. Activated macrophages express tumor necrosis factor alpha (TNFα) that stimulates naïve CD4^+^ T helper cells. Differentiated T helper cells produce interleukin-2 (IL-2) and interferon (IFN)-g, which respectively enhance cell proliferation and CD8 cytotoxic T cell activity. Also, sarcoidosis granulomas are inhibited by T helper 17-polarized effector T cells, whose presence seems to influence the course and severity of the disease [4].

The possible phenotypic manifestations and their relative approximate frequency in NS are presented in Table 1, whereas in Table 2, potential diagnostic features of NS are enlisted.

**Table 1:** Possible phenotypic manifestation of NS and relative approximate frequency

Clinical manifestation	Frequency	Description
Cranial neuropathy Optic nerve Facial nerve Vestibulocochlear nerve	50–75% 7–35% 11–25% 3–17%	Granulomatous infiltration of cranial nerve nuclei or nerves can produce cranial neuropathy; a subacute and progressive course is typical, and a bilateral involvement is common. Heerfordt syndrome (parotitis, facial nerve palsy, low-grade fever, and anterior uveitis) is a rare manifestation MRI often reveals leptomeningeal gadolinium enhancement nearby nerve emergency
Leptomeningitis (pachymeningitis)	10–20% (rare)	The involvement of most commonly leptomeninges, rarely pachymeninges, may determine a subacute meningitis syndrome that can persist, becoming a chronic meningitis Radiologically, it is possible to observe lepto- or pachymeningeal gadolinium enhancement, which is often associated with a nodular component
Myelopathy	5–25%	The disease can affect the spinal cord via several mechanisms, including direct infiltration of spinal cord parenchyma, leptomeninges, or extraspinal tissue with compression of the spinal cord Characteristic imaging findings include nodular and linear leptomeningeal contrast enhancement, eventually associated with intraparenchymal T2 hyperintensity. Longitudinally extensive myelitis (LETM; ≥ 3 segments) is relatively common
Parenchymal disease Mass lesion Encephalopathy	> 50% 5–10% 5–10%	Intraparenchymal mass-like lesions develop in roughly 15% of cases and can produce seizures, focal deficit, and neuropsychiatric illness. MRI findings include T2 hyperintense and T1 isointense lesions that may or may not enhance Nonspecific with matter T2/FLAIR hyperintense lesions without gadolinium enhancement are common and may be small and focal or larger, more diffuse lesions that resemble chronic vascular disease
Hydrocephalus	10%	
Sellar disease	2–8%	Hypothalamic/pituitary involvement mostly determine anterior hypopituitarism (LH/FSH 89%, TSH 67%, GH 50%, and ACTH 49%), diabetes insipidus (65%), and hyperprolactinemia (49%) MRI findings include thickening and contrast enhancement of the pituitary gland or stalk some
Peripheral neuropathy	2–86%	NS can be associated with both large and small fiber polyneuropathies or polyradiculoneuropathies with pure motor, sensory, or mixed sensorimotor features, including a Guillain–Barré-like syndrome. Symmetric chronic sensorimotor neuropathy with axonal features on electromyography (EMG) is the most common presentation. It also may be observed a mononeuritis multiplex with axonal features. Biopsy confirmation is required to support an eventual association of neuropathy with sarcoidosis is best
Vascular disease	Rare	Ischemic and hemorrhagic stroke rarely occur in patients with NS in case of small, medium, or large vessel vasculitis or phlebitis, a vascular compression from mass-like lesion (that can also determine venous sinus thrombosis)

**Table 2:** Potential diagnostic features of NS

Serum testing	Hypervitaminosis D and hypercalcemia/hypercalciuria occasionally present Serum angiotensin converting enzyme (ACE) levels are elevated in up to 60% of cases Both these findings showed low sensitivity and specificity
Cerebrospinal fluid (CSF) analysis	Mild/moderate pleocytosis with lymphocyte predominance and elevated protein is typical; particularly in the acute phase, neutrophiles may be present CSF protein elevation is a nonspecific marker of neuroinflammation; oligoclonal bands and elevated immunoglobulin G (IgG) index are found in 20–40% of cases In one study, CD4/CD8 ratio and IL-6 concentration were elevated in NS compared with multiple sclerosis patients None of the above-mentioned findings are specific for NS
MRI	MRI with and without gadolinium is the most appropriate imaging modality. Gadolinium enhancement is a marker of active disease. The findings differ accordingly to clinical presentation, as described in Table 1

According to the current diagnostic criteria from the 2018 Neurosarcoidosis Consortium Consensus [5], a definite diagnosis of NS requires a biopsy confirmation in the central or peripheral nervous system. However, the neuroanatomic localization often precludes biopsy, and it is necessary to investigate evidence of systemic sarcoidosis to establish histopathological confirmation of the underling illness. About half of the cases of central nervous system (CNS)-predominant sarcoidosis have an abnormal chest X-ray at the time of diagnosis [6]. In negative cases, chest, abdominal, and pelvic computed tomography (CT) with intravenous (IV) contrast is recommended. If structural imaging does not reveal any potential biopsy target, combined fluorodeoxyglucose positron emission tomography (PET)/CT scan may reveal active lymph nodes or others occult lesions [7].

Corticosteroids remain the mainstay of acute treatment: initial dosing of prednisone 1 mg/Kg/die is suggested in presence of CNS abnormalities, whilst prednisone 0.5 mg/kg/die may be sufficient in case of cranial and or peripheral neuropathy. Nonresponders or patients affected by severe disease at presentation may benefit from course of a 3–5-day intravenous methylprednisolone 1 g/die [8, 9]. Patients with NS require in all cases a prolonged course of glucorticoids therapy (at least 6–12 weeks) and progressive tapering. Despite that, a significant portion of patients with NS will be refractory to glucocorticoids or will experience relapse of disease activity when attempting to taper down steroids [2]. In such cases, steroid-sparing immunosuppressive agents become necessary. Various steroid-sparing agents have been proposed in NS, such as methotrexate, azathioprine, cyclophosphamide, and mycophenolate mofetil. In the absence of further clinical studies, the choice is still based on clinical judgment, considering comorbidities and adverse effects.

In terms of second line steroid-sparing treatments, the best studied in NS is infliximab, a chimeric monoclonal antibody to TNFα which appears to be able to inhibit the formation of granulomas in sarcoidosis and induce apoptosis via complement-dependent and antibody-dependent cytotoxicity [2, 3, 10]. A multi-institutional study showed a favorable clinical response in 77% of patients and a magnetic resonance imaging (MRI) response in 82% of patients treated with infliximab [3]. The anti-IL6 monoclonal antibody tocilizumab has also shown efficacy in refractory sarcoidosis treatment with pulmonary, sinus, and cutaneous involvement [11]. Treatment is generally targeted to remission and often continued for a few to several years. After treatment discontinuation, it is still important to monitor the patient clinically and radiologically for recurrence, which tends to occur as early as 3–6 months after discontinuation.

## Case presentation

A 29-year-old Caucasic male presented to neurological evaluation with a 2-month history of episodic vertigo associated with tachycardia and intense sweating, especially in concomitance with head rotatory movements and postural changes. In addition, 2 weeks from admission, he experienced mild cervical pain and bilateral auricular fullness. He underwent ear–nose–throat (ENT) evaluation, which revealed a bilateral audiometric perceptive deficit of acute tones; a 7-day course of oral prednisone, a brain MRI with and without gadolinium, and a further neurological evaluation were prescribed. The brief course of oral prednisone ameliorated the clinical symptoms. Brain MRI revealed multiple lesions, many of them showing contrast enhancement. The neurological examination revealed mild left ataxic hemisyndrome. The patient was admitted to ward for further investigations. He reported a medical history of asymptomatic Mediterranean anemia. Family history was negative. MRI of the brain and spinal cord with and without gadolinium showed left thalamocapsular/periventricular, right fronto-opercular white matter, left middle cerebellar peduncle T2-FLAIR hyperintense lesions, nodular leptomeningeal contrast enhancement along the skull base and the spinal cord, and enhancement of the II–III cranial nerves and the cauda equina. Lab analysis revealed a negative thrombophilia and autoimmune panel, reduced levels of angiotensin-converting enzyme (ACE), no hypercalcemia, and no hypercalciuria. A blood lymphocyte typing evidenced a mild lymphopenia and a high CD4/CD8 ratio (2.67). Cerebrospinal fluid analysis showed a moderate pleocytosis with lymphocyte predominance and elevated proteins, oligoclonal bands profile 3, elevated immunoglobulin G (IgG) index, and negative microbiological testing including tuberculosis and West Nile polymerase chain reaction (PCR); the cytofluorimetric analysis of the lymphocytes revealed a T-cell predominance (65%) with elevated CD4/CD8 ratio (CD4 85%/CD8 15%) in absence of monoclonality. Body imaging (chest, abdomen, and pelvis PET/CT scan) revealed lung and mediastinal lymphadenopathies. Samples of the affected lymph nodes were obtained through bronchoscopy, and the histological examination revealed noncaseating granulomatous inflammation.

After pneumologist and rheumatologist consultations, according to the 2018 Neurosarcoidosis Consortium Consensus diagnostic criteria, a diagnosis of probable NS was made. An oral therapy with 50 mg/die prednisolone was started with complete clinical recovery and discharge from the ward.

**Fig. 1:**
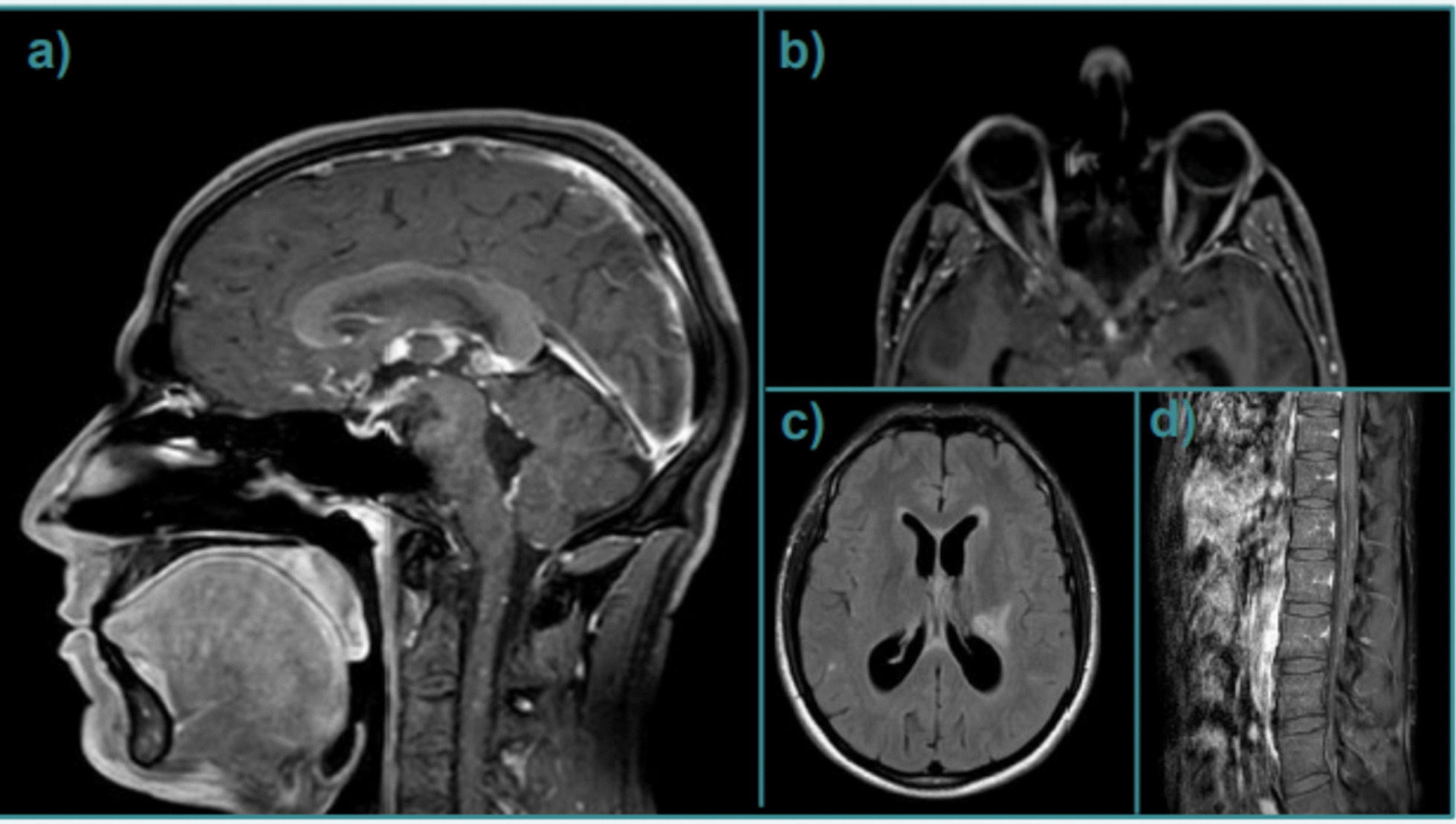
Evidence of nodular contrast enhancement of the leptomeninges (**a**), optic nerves (**b**), white matter lesions (**c**), and cauda equina (**d**)

**Fig. 2:**
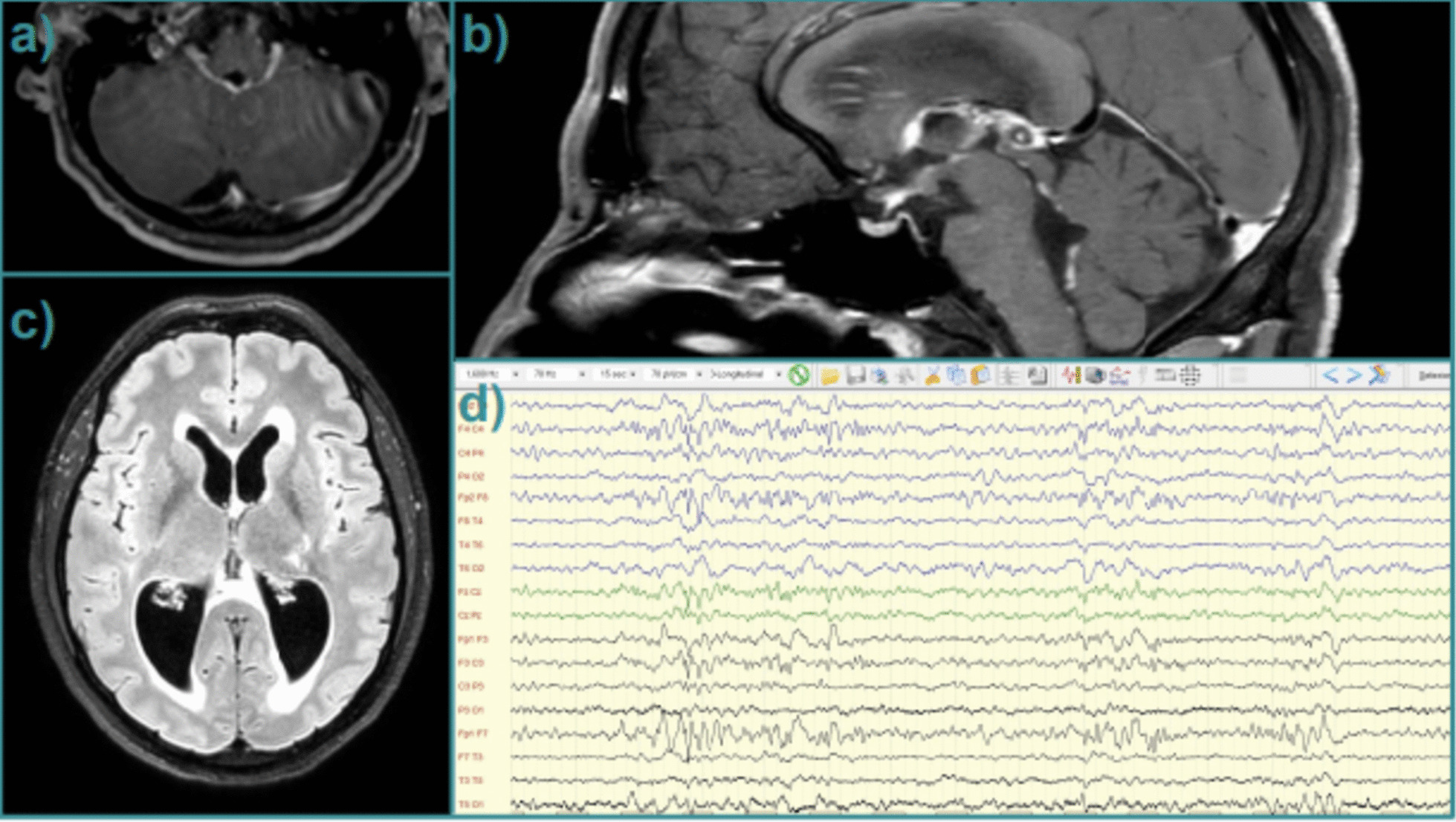
Main examinations performed after clinical worsening; brain MRI shows biventricular hydrocephalus and contrast enhancement of ependyma at the cerebral aqueduct (III and IV ventricles, **a**-**c**). Electroencephalogram shows intercritical epileptic discharges (**d**)

At a 1-month follow-up evaluation, prednisone was tapered to 37.5 mg/die. In the weeks following the decalage, the patient developed intense headaches and slowdown. The follow-up neurological examination resulted negative. A 3T brain MRI showed lateral ventricular dilatation, periventricular seepage, and contrast-enhancement of the ependyma at Monro, Luschka foramina, and cerebral aqueduct. The patient underwent ophthalmologist evaluation with evidence of bilateral papillary edema and enlargement of the dead spot. Again, he was readmitted, and in the first day of hospitalization, a generalized epileptic seizure was reported and treated with intravenous diazepam; a standard electroencephalography documented left frontal sporadic epileptic abnormalities (Figs. 1, 2). Prompt administration of a 5-day course of intravenous 1 g methylprednisolone, acetazolamide, and levetiracetam was followed by a rapid clinical recovery sparing neurosurgical intervention. After the acute phase, subsequent oral prednisolone 75 mg/day and methotrexate 15 mg/week were started. Following serial MRI controls of brain and spinal cord, with and without gadolinium, documented progressive reduction of the lateral ventricle dilatation and of the periventricular seepage; absence of contrast-enhancement at the foramina of Monro and Luschka and minimal residual contrast-enhancement at the cerebral aqueduct were documented. The nodular leptomeningeal, cranial nerves II and II, and cauda equina enhancement remained stable; the T2-FLAIR hyperintense brain lesions were less visible. New ophthalmologist evaluation documented bilateral papillary atrophy with mild enlargement of the blind spot; new standard electroencephalography revealed a quantitative and qualitative reduction of the epileptic abnormalities. Given radiological recovery and clinical stability, the patient was discharged.

After 1 month, a new ophthalmological evaluation revealed reduction of the enlargement of the dead spot, and a new MRI of the brain and spinal cord, with and without gadolinium, documented a further reduction of the ventricular and cerebral aqueduct dilatation and of the periventricular seepage, as well as absence of contrast-enhancement at the cerebral aqueduct. At the neurological evaluation the patient resulted asymptomatic, and the neurological examination was negative for focal deficits. A progressive tapering of oral prednisolone was started to maintain only methotrexate as long-term immunosuppressive treatment. Accordingly, acetazolamide was progressively tapered and levetiracetam maintained. The medical treatment ensured prolonged clinical and radiological stability.

## Discussion and conclusion

Hydrocephalus is reported in approximately one-tenth of patients with NS [12]. It is generally noncommunicating and caused by obstruction of the outflow from the fourth ventricle and/or the cerebral aqueduct due to the presence of sarcoidotic granulomas. The onset may be either acute or subacute, and the presenting symptoms are related to increased intracranial pressure. It represents a potentially fatal manifestation that requires a prompt initiation of treatment. In a recent literature review, it has been reported as a mortality rate of 22% [12], where multiple articles reported a sudden death. In another literature review, there was an even higher mortality of 75% in patients with NS who presented with seizures and hydrocephalus [13].

High-dose intravenous corticosteroid treatment must be considered in all cases of NS hydrocephalus to reduce granulomatous inflammation. An intensification of immunosuppressive treatment is required in case of a relapse, as a persistent or recurring granulomatous chronic meningitidis can lead to an additional site of obstruction throughout obstruction of the cerebrospinal fluid (CSF) shunt [14]. An additional treatment neurosurgical intervention may be considered, such as CSF shunting, ventriculostomy, or decompression. However, neurosurgery can lead to severe complications, and it is not always definitive: a reintervention can be required because of failure of a CSF shunt or ventriculostomy. Of 36 cases reported in literature, only 7 (19%) were treated with immunosuppressant therapy alone; in all other cases a neurosurgical treatment was performed (CSF shunting, ventriculostomy, and other interventions). A total of 31 (89%) out of 36 patients underwent corticosteroid therapy. Second-line treatment consisted of azathioprine and methotrexate in 4 (11%) out of 35 patients. Third-line treatment with infliximab was started in two (6%) patients. An improvement was observed in 27 (75%) of patients, deterioration was observed in 1 (3%), and deterioration leading to death was observed in 8 (22%) patients. The causes of death were cerebral herniation, intractable seizures, pulmonary embolism, and neurosurgery complications [15].

Hydrocephalus is a severe potential complication of NS, associated with high mortality. In most cases reported in literature, neurosurgical treatment was associated to medical treatment. However, neurosurgery can lead to severe complications; therefore, further studies are needed to identify the baseline characteristic by which a patient should be addressed to neurosurgical management in addition to medical therapy.

## Data Availability

Not applicable.
